# MicroRNA Expression Differences in Blood-Derived CD19+ B Cells of Methotrexate Treated Rheumatoid Arthritis Patients

**DOI:** 10.3389/fimmu.2021.663736

**Published:** 2021-04-09

**Authors:** Fatima Heinicke, Xiangfu Zhong, Siri T. Flåm, Johannes Breidenbach, Magnus Leithaug, Marthe T. Mæhlen, Siri Lillegraven, Anna-Birgitte Aga, Ellen S. Norli, Maria D. Mjaavatten, Espen A. Haavardsholm, Manuela Zucknick, Simon Rayner, Benedicte A. Lie

**Affiliations:** ^1^ Department of Medical Genetics, Oslo University Hospital and University of Oslo, Oslo, Norway; ^2^ Norwegian Institute for Bioeconomy Research, National Forest Inventory, Ås, Norway; ^3^ Division of Rheumatology and Research, Diakonhjemmet Hospital, Oslo, Norway; ^4^ Department of Rheumatology, Martina Hansens Hospital, Bærum, Norway; ^5^ Department of Biostatistics, Oslo Centre for Biostatistics and Epidemiology, University of Oslo, Oslo, Norway

**Keywords:** miRNA, microRNA, rheumatoid arthritis, CD19+ B cells, methotrexate, NGS

## Abstract

Rheumatoid arthritis (RA) is a complex disease with a wide range of underlying susceptibility factors. Recently, dysregulation of microRNAs (miRNAs) in RA have been reported in several immune cell types from blood. However, B cells have not been studied in detail yet. Given the autoimmune nature of RA with the presence of autoantibodies, CD19+ B cells are a key cell type in RA pathogenesis and alterations in CD19+ B cell subpopulations have been observed in patient blood. Therefore, we aimed to reveal the global miRNA repertoire and to analyze miRNA expression profile differences in homogenous RA patient phenotypes in blood-derived CD19+ B cells. Small RNA sequencing was performed on CD19+ B cells of newly diagnosed untreated RA patients (n=10), successfully methotrexate (MTX) treated RA patients in remission (MTX treated RA patients, n=18) and healthy controls (n=9). The majority of miRNAs was detected across all phenotypes. However, significant expression differences between MTX treated RA patients and controls were observed for 27 miRNAs, while no significant differences were seen between the newly diagnosed patients and controls. Several of the differentially expressed miRNAs were previously found to be dysregulated in RA including miR-223-3p, miR-486-3p and miR-23a-3p. MiRNA target enrichment analysis, using the differentially expressed miRNAs and miRNA-target interactions from miRTarBase as input, revealed enriched target genes known to play important roles in B cell activation, differentiation and B cell receptor signaling, such as *STAT3*, *PRDM1* and *PTEN.* Interestingly, many of those genes showed a high degree of correlated expression in CD19+ B cells in contrast to other immune cell types. Our results suggest important regulatory functions of miRNAs in blood-derived CD19+ B cells of MTX treated RA patients and motivate for future studies investigating the interactive mechanisms between miRNA and gene targets, as well as the possible predictive power of miRNAs for RA treatment response.

## Introduction

Rheumatoid arthritis (RA) is a systemic autoimmune disorder associated with chronic inflammation, mainly in the joints, causing bone erosion and loss of mobility if not treated effectively at an early disease stage. The first-line treatment in RA is the disease-modifying antirheumatic drug methotrexate (MTX) (1). However, a majority of RA patients do not respond toward MTX monotherapy (2–4), and no biomarker exists to reliably predict the success or failure of MTX treatment for a given patient.

Even though the precise pathogenesis of RA is unknown, lymphocytes, such as B cells, have been reported to play a major role. Auto-antibodies can be detected in serum of RA patients up to 10 years prior to diagnosis (5, 6). Furthermore, B cells are major producers of chemokines and cytokines and can thereby influence RA pathogenesis (7). Disturbances in CD19+ B cell subpopulations have been observed in peripheral blood of RA patients, indicating breaks in tolerance within B cell development potentially leading to self-reactive B cells (8, 9).

Cell phenotypes are controlled by gene regulatory mechanisms, and microRNAs (miRNAs) have been found to be key regulatory molecules during B cell development and maturation (10–12). MiRNAs are small non-coding RNAs (~22 nucleotides) that regulate gene expression post-transcriptionally. Dysregulated miRNA signatures have also been demonstrated to be linked to the pathogenesis of several diseases (reviewed by Li and Kowdley (13)). The first dysregulated miRNA in RA was found when comparing joint tissue from RA and osteoarthritis patients (14). Since then, numerous studies investigating miRNA expression levels in RA have been performed, mainly in synovial joint tissue (14, 15) but also in serum (16), peripheral blood (16, 17), peripheral blood mononuclear cells (PBMC) (15, 17), blood-derived CD4+ T cells (15, 18) and specific CD4+ T cell subsets (19). However, studies investigating miRNAs in RA CD19+ B cells from blood are lacking. To the best of our knowledge, only one study has been published (20), but this study of blood-derived CD19+ B cells solely investigated the expression of miR-155-5p.

The aims of our study were to assess the global miRNA repertoire of peripheral blood CD19+ B cells, by high throughput sequencing, and to uncover miRNA expression differences between homogenous RA patient phenotypes (newly-diagnosed and MTX treated) and healthy controls.

## Material and Methods

### Patient and Healthy Control Subjects

**Figure 1:**
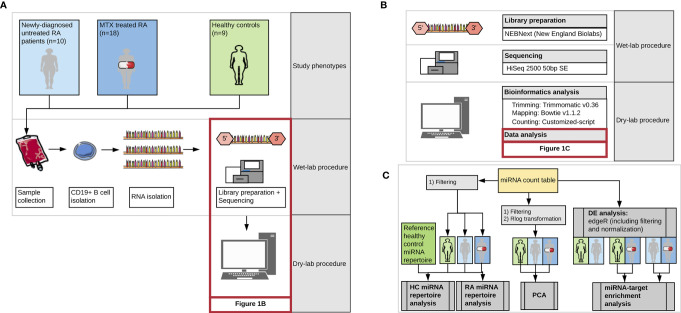
Workflow of the study representing **(A)** the three study phenotypes and major steps during wet-lab and computational analysis, **(B)** detailed representation of the kit, technology and tools used during miRNA library preparation, sequencing and bioinformatics analysis, and **(C)** detailed presentation of the steps included in the data analysis. DE, differential expression analysis; MTX, Methotrexate; HC, healthy controls. Images from Servier Medical Art (Servier. www.servier.com, licensed under a Creative Commons Attribution 3.0 Unported License) were used in the figure.

An overview of our study design is presented in **Figure 1**. Twenty-eight patients diagnosed with RA according to the 2010 Rheumatoid Arthritis classification criteria (21), recruited from the Diakonhjemmet Hospital (n=27) and Martina Hansen’s Hospital (n=1) were included in the study. The patients were recruited from two clinical cohorts: (i) the Norwegian Very Early Arthritis Clinic (NOR-VEAC) observational study (ISRCTN05526276) and (ii) The Assessing Withdrawal of Disease-Modifying Anti-rheumatic Drugs in Rheumatoid Arthritis (ARCTIC REWIND) trial (ClinicalTrials.gov identifier NCT01881308). RA patients belonging to the NOR-VEAC cohort (n=10) were, at the time of inclusion, newly-diagnosed and had an active ongoing disease, but MTX or prednisolone had not yet been initialized (hereinafter referred to as newly-diagnosed RA patients). The patients belonging to the ARCTIC REWIND trial (n=18) had both received unchanged MTX as monotherapy and had additionally been in stable remission for at least one year. At the time of inclusion, the disease activity score (DAS) was less than 1.6 and the patients had no swollen joints in any of the 44 assessed joints. The disease duration was less than four years and the time since diagnosis is approximately equivalent to duration of MTX treatment. This patient group is hereinafter referred to as MTX treated RA patients. All patients were clinically examined and parameters such as anti-citrullinated peptide antibodies (ACPA), rheumatoid factor (RF) status, C-reactive protein (CRP), erythrocyte sedimentation rates (ESR), DAS and DAS28 were recorded (**Supplementary Table 1**). Age, gender and smoking-status matched healthy controls (n=9) were recruited from the Norwegian Bone Marrow Donor registry. We refer to newly-diagnosed, MTX treated RA patients and healthy controls as study phenotypes.

### Ethics

Written informed consent was voluntary given by all patients. The study was approved by the Norwegian National Health Authorities and Regional Ethics Committee (REK 2015/1546).

### Blood Sample Collection and Immune Cell Isolation

At the time of inclusion and clinical assessment, we also collected 200 ml whole blood using blood bags (Fresenius Kabi, Oslo, Norway) containing 5 mM EDTA (Thermo Fisher Scientific Inc, Massachusetts, USA) as an anticoagulant. The blood was processed within 30 minutes after collection by diluting it 2:3 in PBS (PBS without MgCl, Thermo Fisher Scientific) with 1 mM EDTA and 2% FCS (BioNordika, Oslo, Norway). For isolating PBMCs, the blood:PBS mixture was layered on top of 14 ml Lymphoprep in SepMate™ tubes (Stemcell Technologies, Vancouver, Canada) and centrifuged at 1200 x g for 10 minutes at room temperature with the brake on. The harvested PBMCs were washed once with PBS containing 2 mM EDTA and were immediately used for cell isolation. CD19+ B cells were magnetically isolated using the EasySep Human CD19 positive selection kit (Stemcell technologies) according to the manufacturer’s protocol. Flow cytometry, using a BD Accuri C6 (BD Biosciences, San Jose, USA), was used to assess the purity and viability of the isolated cell types by staining the cells with desired antibodies.

### RNA Isolation

RNA was isolated using the RNA/DNA/Protein Purification Kit (Norgen Biotek, Ontario, Canada) according to the manufacturer’s protocol. The starting material was in the range of 1 x 10^6^ to 5 x 10^6^ cells. In the lysis step, a mixture of beta-mercaptoethanol and Buffer SKP was used in a 1:10 ratio. Genomic DNA was removed using the RNase-free DNase I kit (Norgen Biotek). RNA integrity was analyzed on a 2100 Bioanalyzer (Agilent Technologies, California, United States), and the concentration was measured on a Qubit 2.0 fluorometer (Thermo Fisher Scientific, Massachusetts, United States). RNA integrity values were 8 or above for all samples (**Supplementary Table 2**).

### miRNA Library Preparation and Sequencing

The small RNA libraries were produced using the NEBNext Multiplex Small RNA Library Prep kit (New England Biolabs, Massachusetts, USA) according to the manufacturer’s protocol. The protocol consists of the following steps: 3’ adapter ligation, hybridization of reverse transcriptase primer, 5’ adapter ligation, reverse transcription and PCR. Given the total RNA starting material of 100 ng, the 3’ adapter, reverse transcriptase primer and 5’ adapter were diluted 1:2 in nuclease free water as recommended in the protocol. For the PCR, 15 cycles were used. The small RNA library constructs were purified using AMPure XP beads (Beckman Coulter Life Sciences, Indianapolis, USA) in a 1:3.2 ratio. The presence of small RNA library constructs was verified using the TapeStation 2200 High Sensitivity D1000 kit (Agilent Technologies) by checking for the presence of a miRNA peak at 143-146 bp. Unwanted fragments and adapter dimers were removed using Pippin Prep (Sage Science, Massachusetts, USA) and 3% Agarose Gel Pippin Cassettes. The procedure was optimized to harvest only fragments ranging between 130 bp and 160 bp. The final size and yield of the miRNA libraries were measured using the Bioanalyzer 2100 system (Agilent Technologies).

Per flow cell lane, 16 miRNA libraries were multiplexed. The libraries were sequenced on single-read flow cells of a HiSeq2500 (Illumina, California, USA) for 50 cycles.

### Bioinformatics Analysis

The 3’ adapters from the demultiplexed samples were trimmed using Trimmomatic v0.36 (22) with the options SE, ILLUMINACLIP=adapter.fa:2:30:7 and the following adapter sequence: AGATCGGAAGAGCACACGTCT. The remaining reads after trimming were mapped to a manually curated reference set based on the human mature miRNA sequences specified in miRBase v22 (23). The curations applied have been described previously by Zhong, Heinicke (24) and enable unambiguous mapping. Briefly, independent miRBase v22 miRNA entries presenting the same mature miRNA sequence were collapsed into one entry. Furthermore, entries with miRNA sequences completely overlapping other miRNA sequences, just being shorter, were deleted. These filtering steps resulted in a reference set called *miRBasev22FH* containing 2615 mature miRNA sequences. Read mapping was performed using bowtie v1.1.2 (25) without allowing for mismatches. A customized script was used to count mapped miRNA reads, which perfectly matched entries in the *miRBasev22FH* reference set.

### Data Analysis

Data and statistical analyses were performed using R v3.5.2 (26). Unless stated otherwise, ggplot2 (27) was used for visualization.

For the miRNA repertoire analysis, upset plots were produced using the R package UpSetR v1.4.0 (28). For each study phenotype, only miRNAs with an expression of at least 100 count per million (CPM) in at least one third of the investigated samples were included in the analysis. Our observed miRNA repertoire of CD19+ B cells was further compared to previous studies. Because data on global blood-derived CD19+ B cell miRNA expression profiles in RA patients have not been previously published, we focused on studies performed on healthy controls (29–33). As variation in the experimental procedures, such as different lymphocyte isolation methods or miRNA expression profiling techniques, will influence the observed miRNA repertoire (34, 35), only the study by Juzenas, Venkatesh (29) was deemed comparable.

Principal component analysis (PCA) was performed using the R function prcomp() for assessing the global miRNA expression signatures. The first two principal components of the miRNA read count matrix were used and plots were generated using the function plotPCA() from the Bioconductor package DESeq2 1.20.0 (36). For the PCA, the miRNA read count matrix was (i) filtered for low read counts with a threshold of 100 CPM in at least one third of the overall study population and (ii) regularized log (rlog) transformed using the homonymous function implemented in DESeq2.

Differential expression analysis using the Bioconductor package edgeR v3.22.3 (37) was performed by comparing the following study phenotype pairs: (i) healthy controls and newly-diagnosed patients, (ii) healthy controls and MTX treated RA patients and (iii) newly-diagnosed and MTX treated RA patients. Prior to the analysis, low expressed miRNAs were filtered out. The threshold was set to 100 CPM in one third of the study phenotype pairs. This ensured miRNAs undetected in the miRNA repertoire analysis to be included in the differential expression analysis. The filtered read counts were normalized using the default normalization method, trimmed mean of M, implemented in edgeR. We used the quasi-likelihood F-test for testing for differentially expressed miRNAs. After adjusting for multiple testing using the method of Benjamini and Hochberg for controlling the false discovery rate (FDR), a miRNA was defined as significantly differentially expressed between study phenotype pairs when the corresponding adjusted p-value was lower than 0.05.

The web-based tool Mienturnet v1 was used for miRNA-target enrichment analysis (38). As input to the analysis the differentially expressed miRNAs from the previous step were used. Additionally, we chose miRTarBase v.7.0 containing experimentally validated miRNA-target interactions as input for the analysis. The threshold for the minimum number of miRNA-target interactions was set to three and the adjusted p-values to 0.05. Only enriched miRNA-target interactions, which were previously annotated with strong experimental evidence (e.g. Western blot or Luciferase assays), were included for the subsequent network visualization.

Genes resulting from the enrichment analysis (enriched target genes) found to be targeted by at least three of our differentially expressed miRNAs were investigated for co-expression using Immuno-Navigator (39). Cell type specific co-expression of genes were tested in one of the available CD19+ B cells (mature B cells), as well as in other immune cell types for comparisons using the default parameters.

The genome wide association studies catalogue (40) was searched (November 2020) for autoimmune diseases reported to be significantly associated (p<5x10^-8^) with the genes encoding the miRNA target mRNAs.

## Results

### Characteristics of the Study Phenotypes

Demographic and clinical characteristics of the patients and healthy controls are summarized in **Table 1**, and presented for each individual in **Supplementary Table 1**. No significant (p>0.05) differences were seen in age, gender or smoking status between the newly-diagnosed (treatment naïve) or MTX treated RA patients compared to healthy controls. The inflammatory markers ESR and CRP, as well as the DAS28, were elevated in the newly-diagnosed compared to the MTX treated RA patients. The majority of patients in both RA cohorts were ACPA and RF positive.

**Table 1:** Summary of demographic and clinical characteristics in the study phenotypes.

	Newly-diagnosed RA patients (n=10)	MTX treated RA patients (n=18)	Healthy controls(n=9)
Female [in %]	8 [80]	15 [83.3]	7 [77.8]
Age of recruitment (median, [range])	57.5, [36-73]	46, [25-70]	53, [38-65]
Smoking [in %]	7 [70]	13 [72.2]	6 [66.7]
ACPA positive [in %]	9 [90]	14 [77.8]	NA
RF positive [in %]	8 [80]	12 [66.7]	NA
DAS28 (median, [range])	5.05, [2.2-8.7]	1.35, [1-2.3]	NA
CRP in mg/L (median, [range])	15.5, [4-108]	1, [1-10]	NA
ESR in mm/h (median, [range])	30, [18-68]	7, [3-26]	NA
Time since diagnosis in Years* (median, [range])	0	3, [2-4]	NA

NA, not applicable; MTX, Methotrexate; ACPA, anti-citrullinated peptide antibodies; RF, rheumatoid factor status; DAS28, disease activity score 28; CRP, C-reactive protein; ESR, erythrocyte sedimentation rates.

*Time since diagnosis in years is approximately equivalent to the duration of MTX treatment.

### CD19+ B Cell Purity and Sequencing Data Summary

CD19+ B cells were isolated from peripheral blood using magnetic cell sorting. Flow cytometry analysis showed that the median purity of the isolated cells was 91% (range: 74% - 98%). The ensuing total RNA isolation and small RNA sequencing generated 576 million (M) raw reads with a median sequencing depth of 15.5 million (M) raw reads per sample (range: 6.3 M to 27.3 M reads per sample, **Supplementary Table 3**). Of those, 573 M reads remained after adapter trimming. Mapping the trimmed reads to our manually curated miRNA reference set and subsequent counting revealed that more than 45 M reads belonged to miRNA sequences which corresponded to on median 1.2 M reads per sample (range: 0.5 M - 2.9 M, **Supplementary Table 3**).

### CD19+ B Cell miRNA Repertoires

**Figure 2:**
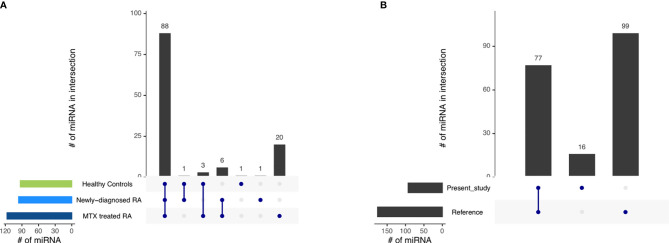
The miRNA repertoire observed in blood-derived CD19+ B cells **(A)** across the different study phenotypes and **(B)** comparing the miRNA repertoire of healthy controls in the present study with those of a reference dataset (29). Interactions are indicated by the blue dots and lines. The number of miRNAs in the intersection is represented by the black bars.

First, we investigated which miRNAs could be detected within the study phenotypes and thereby form the miRNA repertoire within the CD19+ B cell sample set. After filtering out lowly expressed miRNAs (<100 CPM in one third of each study phenotype), 93, 96 and 117 miRNAs were detected in the healthy controls, newly-diagnosed and MTX treated RA patients, respectively. Of these miRNAs, 88 miRNAs were detected in all three study phenotypes (**Figure 2A**, **Supplementary Table 4**). A closer investigation of the 32 miRNAs not detected across all phenotypes revealed that all of them were in fact expressed but below the filtering threshold applied.

Next, we compared our healthy control CD19+ B cell miRNA repertoire to a published miRNA repertoire of CD19+ B cells from healthy individuals as a reference (29). The majority of miRNAs reported by us were also detected in the reference (**Figure 2B**, **Supplementary Table 5**). Additionally, we observed 16 miRNAs uniquely represented in our dataset, while 99 miRNAs reported in the reference were not annotated in our control dataset. As a lower filtering threshold was applied in the reference compared to our study, we reduced the filtering threshold from 100 to 5 CPM in one third of the healthy controls for this specific comparison. By doing so, 63 of the 99 previously undetected miRNAs could be additionally detected also in our data set (**Supplementary Table 6**).

### Global miRNA Expression Signatures Are Partly Influenced by RA Phenotype

**Figure 3:**
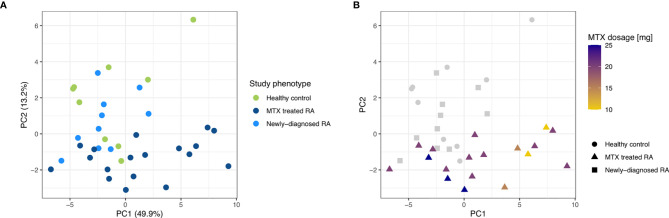
PCA plots for the first two principal components on low read count filtered and rlog transformed miRNA read counts of the three study phenotypes. **(A)** The study phenotypes are indicated by different colors. The percentage of variance explained by the principal component is indicated. **(B)** MTX dosage is indicated by different colors while different shapes were used to differentiate the study phenotypes.

After establishing the similarities in miRNA repertoires among the CD19+ B cells from the three study phenotypes, we next addressed miRNA expression levels to explore phenotypic global expression signatures. A PCA revealed that more than 63% of the total variation in the data is explained by principal component (PC) 1 and PC2. This value increases to more than 80% when investigating the first 5 PCs. The PCA plot shows that the first two PCs did not allow a complete separation of the three study phenotypes. The MTX treated RA patients had a higher within group variance in PC1 compared to the other two study phenotypes. In addition, the MTX treated patients tended to separate from the newly-diagnosed RA patients and controls (**Figure 3A**). Notably, the MTX treated phenotype also seemed to consist of two clusters. Investigation of the available clinical characteristics revealed that one cluster of MTX treated patients was dominated by RA patients who received lower dosages of MTX (**Figure 3B**). No other clinical characteristic (including DAS28, age, smoking status, gender, antibody status or disease duration) explained the two clusters of MTX treated patients (**Supplementary Figure 1**).

### miRNAs Dysregulated in MTX Treated RA Patients

**Figure 4:**
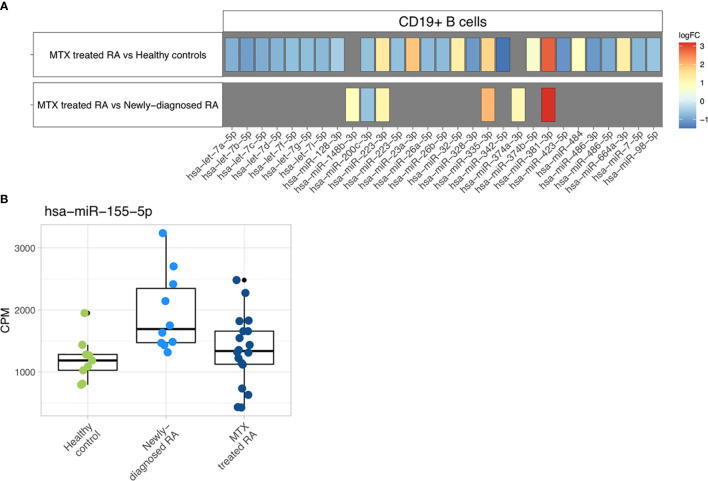
Differential expression analysis. **(A)** Differentially expressed miRNAs are represented with their specific log2 fold changes. **(B)** Boxplots representing the abundance of miR-155-5p in CPM based on study phenotype.

Differential expression analysis of individual miRNAs revealed 27 differentially expressed miRNAs (FDR<0.05) in MTX treated RA patients compared to healthy controls (**Figure 4A**, **Supplementary Table 7**). More downregulated than upregulated miRNAs were observed in the MTX treated RA patients in contrast to the controls. No miRNA was found to be differentially expressed in newly-diagnosed RA patients compared to healthy controls. However, when comparing MTX treated and newly-diagnosed patients, six miRNAs were differentially expressed (**Figure 4A**, **Supplementary Table 7**). Four of these miRNAs had also been found to be differentially expressed in the same direction in MTX treated RA patients when compared to controls.

MiR-155-5p, the only miRNA previously investigated and found dysregulated in blood-derived CD19+ B cells from RA patients (20), was not found at significantly different expression levels in our study population. However, a tendency of higher miR-155-5p expression in the newly-diagnosed RA patient group could be observed (**Figure 4B**).

### miRNA Target Enrichment

**Figure 5:**
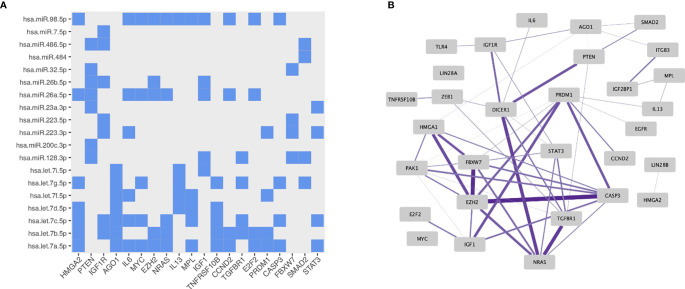
Properties of the miRNA-target enrichment analysis based on the differentially expressed miRNAs identified from comparing MTX treated RA patients to healthy controls: **(A)** Enriched miRNA-target interactions. The x-axis shows the specific enriched target gene and the y-axis the specific targeting miRNA. **(B)** Gene expression correlation network in mature B cells generated using the Immuno-Navigator database. Target genes with correlated expressions are connected by an edge. The thicker and more saturated the line, the stronger the correlation.

Next, the differentially expressed miRNAs (n=29) between MTX treated RA patients compared to either newly-diagnosed RA patients or controls and the experimentally validated miRNA-target interactions from miRTarBase were used as input in the miRNA-target enrichment analysis. No significantly associated enriched target genes for the miRNAs differentially expressed between MTX treated and newly-diagnosed RA patients were revealed after correcting for multiple testing. In contrast, the enrichment analysis identified 1108 enriched target genes for the differentially expressed miRNAs between MTX treated RA patients and healthy controls (FDR<0.05). For building a network of the enriched miRNA-target interactions, the results were filtered to retain only those miRNA-target interactions supported by strong experimental methods such as Western blot (**Supplementary Figure 2**). The top 30 enriched target genes showed interactions to 19 of the differentially expressed miRNAs (**Supplementary Figure 3**). The genes *HMGA2*, *PTEN*, *IGF1R* and *AGO1* were among the enriched target genes with the highest number of miRNAs targeting them (**Figure 5A**). Four of the enriched target genes have been reported to be associated with autoimmune diseases in the GWAS catalogue, i.e. *IL-6* (juvenile idiopathic arthritis), *PRDM1* (systemic lupus erythematosus, inflammatory bowel disease), *IL-13* (psoriasis, inflammatory bowel disease), and *TLR4* (inflammatory bowel disease). When using the top 30 enriched target genes as input into the Immuno-Navigator database, a high degree of co-expression in CD19+ mature B cells was identified (**Figure 5B**). This was in contrast to a lower co-expression in most other immune cell types or PBMC listed in the Immuno-Navigator database (**Supplementary Figure 4**).

## Discussion

In the present study, we investigated the miRNA repertoires and miRNA expression levels in peripheral blood-derived CD19+ B cells from three clinically well-defined and homogenous study phenotypes: (i) newly-diagnosed, untreated RA patients, (ii) MTX monotherapy treated RA patients being in remission for at least one year and (iii) healthy controls.

The detected miRNA repertoires were almost identical across all study phenotypes, with little evidence of phenotype specific miRNAs. In line with this, the global miRNA expression signatures showed no distinct PCA clustering according to study phenotype. The same observation has been reported in another global miRNA expression study in T cells (19). Hence, the involvement of miRNAs in RA is likely due to more subtle quantitative differences, which fits to the notion that RA is a complex and multifactorial disease. Likewise, most genetic risk variants are also seen in unaffected individuals and are located in gene regulatory regions affecting the gene expression quantitatively. Differentially expressed miRNAs may thus represent another regulatory mechanism leading to immune dysregulation.

While there are no previous reported miRNA repertoires in blood-derived CD19+ B cells in RA, the miRNA repertoire we observed in our healthy controls largely overlapped with a reference dataset also consisting of healthy individuals (29). The majority of additional miRNAs detected only by the reference dataset were also detected in our dataset when we decreased the filtering threshold from 100 CPM in one third of the samples to 5 CPM. The remaining miRNAs that could only be detected in either our or the reference dataset are most likely a result of technical variations; e.g. differences in library preparation kits, sequencing depth or low read count filtering thresholds rather than being true biological differences.

In the differential expression analysis we mainly observed differences in miRNA expression levels in the RA patients who were MTX treated and in remission, which could be a consequence of the longer disease duration (> 2 years) or a result of MTX treatment. In whole blood of RA patients, differentially expressed miRNAs have been reported after four months of MTX treatment (41). The authors hypothesized that the MTX-induced production of adenosine could cause the activation of transcription factors influencing the dysregulated miRNAs. Further studies are needed to elucidate to what extent the observed CD19+ B cell miRNA changes are involved in long-term pathogenesis or MTX treatment. Whether any of the observed miRNA expression differences are associated with MTX response cannot be addressed by our study, since our cohort did not include MTX non-responders.

To the best of our knowledge, miR-155-5p is the only miRNA previously investigated in blood-derived CD19+ B cells in RA, and was found, by qPCR, to be significantly increased in both untreated early RA patients (n=27) and long-standing RA patients treated with MTX (n=33) compared to healthy controls (n=9) (20). Furthermore, the expression of miR-155-5p was significantly higher in untreated patients than in MTX-treated patients. A similar tendency, albeit not significant, was observed in our patient groups. The discrepancy between these two studies could be due to different miRNA detection methods or the strong inter-individual expression variation observed in miRNAs (42). Also, statistical power could have influenced our results, as both our patient phenotypes consisted of fewer individuals compared to Alivernini, Kurowska-Stolarska (20).

Several of the differentially expressed miRNAs we observed in CD19+ B cells from MTX responding RA patients, have been reported to be involved in RA development or RA treatment response. Decreased expression of miR-486-3p was observed in serum when at-risk individuals progressed into RA (43). Furthermore, miR-223-3p and miR-23a-3p have been hypothesized to be predictors of TNF-alpha/MTX treatment response (44). In particular, miR-223-3p, which had significantly increased expression in CD19+ B cells from our MTX treated RA patients, has been widely studied and reported to be upregulated in other cell types, i.e. macrophages, monocytes and CD4+ T cells (18, 45, 46), and proposed to regulate the formation of osteoclasts in RA synovium (45). We also observed several members of the let-7 miRNA family with significantly decreased expression levels in our MTX treated RA patients. Let-7 miRNAs have been found to play a central role in the regulation of adaptive immune responses by repressing antibody production in activated B cells (47).

Furthermore, our miRNA-target enrichment analysis revealed several enriched target genes with known roles either in B cell function or RA pathogenesis, among them *IL-6*, *IL-13* and *STAT3*. IL-6 is a highly abundant inflammatory cytokine in RA, and our observed reduction in expression of miRNAs from the let-7 family could potentially be involved in upregulating the *IL-6* translation. IL-6 and other cytokines (e.g. IL-10, IL-21, IL-23, and interferons) activate STAT3 through their receptors on lymphocytes. STAT3 is a transcription factor required in naïve B cells to induce plasma cell formation and for proliferation of responding B cells (48). Low-dose methotrexate, as administrated in RA, has been suggested to act through the suppression of the JAK/STAT pathway and specifically a reduced level of STAT3 was observed (49).

The enriched interactions between miRNA and gene transcripts found by us were bioinformatically predicted and need to be experimentally verified. Interestingly though, the enriched target genes showed co-expression in the mature CD19+ B cell subset and some of them (*IL-6*, *IL-13*, *PRDM1* and *TLR4*) have been reported to be associated genetically with autoimmune diseases. Additionally, the biological relevance of the predicted miRNA and gene transcript target warrants functionally verification, and their role in RA and MTX treatment needs to be explored. Nevertheless, it is encouraging that several of the enriched target genes have important functions in B cell biology. *PRDM1* encodes for a B cell specific transcription factor reported to regulate B cell differentiation (50). The expression of *PRDM1* has been reported to be upregulated in autoreactive peripheral blood-derived CD19+ B cells from RA patients (51), and overexpression of *PRDM1* could promote production of antibodies (reviewed by Wu, Deng (52)). *PRDM1* expression is induced by several cytokines among them IL-6 (reviewed by Kim (53)). However, the regulatory effects of let-7 and miR-223-3p on *PRDM1* have only been studied in various cancer types as by today (54, 55), and their involvement in RA needs to be elucidated.

PTEN, encoded by another of our enriched target gene, is known to play an important role in regulating B cell receptor signaling, B cell activation and differentiation in humans. Defective or reduced *PTEN* expression might result in failed B cell anergy (56). In peripheral blood CD19+ B cells of newly-diagnosed patients with type 1 diabetes, systemic lupus erythematosus and autoimmune thyroid disease reduced levels of *PTEN* and increased expression of *PTEN* regulating miRNAs (miR-21-3p, miR-22-3p and miR-7-5p) have been reported (56, 57). This could indicate that reduced *PTEN* expression might predispose for autoimmunity in general.

In conclusion, we observed several miRNAs that were differentially expressed in MTX-treated RA patients being in remission compared to healthy controls, and these miRNAs are suggested to target genes with important functions in B cell activation, differentiation and antibody production. Our miRNA results warrant replications, particularly in longitudinal studies of newly diagnosed RA patients sampled both before and after MTX treatment.

## Data Availability Statement

The data have been uploaded to the Gene Expression Omnibus database with the accession number GSE168284.

## Ethics Statement

The study was approved by the Norwegian National Health Authorities and Regional Ethics Committee (REK 2015/1546). The patients/participants provided their written informed consent to participate in this study.

## Author Contributions

FH, XZ, SF, SR, MZ, and BL were responsible for the conceptualization and design of the study. MTM, SL, A-BA, EN, MDM, and EH contributed in the recruitment and clinical examination of the patients. FH, SF, and ML were involved in the development and execution of the laboratory procedures. FH, XZ, and SR developed the data analysis pipeline and performed the data analysis. MZ and JB contributed to the statistical data analysis. FH, XZ, and JB were involved in data visualization. FH and BL wrote the manuscript. All authors contributed to the article and approved the submitted version.

## Funding

This work was supported by Helse Sør-Øst Grants [2015034 and 2016122].

## Conflict of Interest

The authors declare that the research was conducted in the absence of any commercial or financial relationships that could be construed as a potential conflict of interest.
