# Supplementary material for: MicroRNA Expression Differences in Blood-Derived CD19+ B Cells of Methotrexate Treated Rheumatoid Arthritis Patients
Source: Front Immunol. 2021 Apr 9;12:663736. doi: 10.3389/fimmu.2021.663736 (PMC8062711; doi:10.3389/fimmu.2021.663736)
Supplement: Supplementary file 1 [file DataSheet_1.zip › Supplementary_figures.docx]

| A |  | B |  |
| --- | --- | --- | --- |
| C |  | D |  |
| E |  | F |  |
| G |  | H |  |
| I |  | | |

Figure 1: PCA plots for the first two principal components on low read count filtered and rlog transformed miRNA read counts of the three study phenotypes. Different colors were used to indicate (A) age, (B) gender, (C) smoking status, (D) DAS28 score, (E) ACPA status, (F) RF status, (G) CRP values, (H) ESR values and (I) disease duration in years. Grey color indicates that the clinical parameter was not measured for the specific individual.

Figure 2: Network of enriched miRNA-target interactions. Orange rectangles represent miRNAs and purple rectangles target genes.

| A |  |
| --- | --- |
| B |  |

Figure 3: Properties of the miRNA-target enrichment analysis based on the differentially expressed miRNAs identified from comparing MTX treated RA patients to healthy controls: (A) The top 30 enriched target genes. The x-axis shows the specific target gene and the y-axis presents the number of miRNAs targeting them. (B) Enriched miRNA-target interactions. The x-axis shows the specific target gene and the y-axis the specific targeting miRNA.

| A |  | B |  |
| --- | --- | --- | --- |
| C |  | D |  |
| E |  | F |  |
| G |  |  |  |

Figure 4: Gene expression correlation network. Target genes with correlated expression in (A) CD4+ T cells, (B) CD8+ T cells, (C) macrophages, (D) neutrophils, (E) monocytes, (F) conventional dendritic cells and (G) PBMC are connected by an edge. The thicker and more saturated the line, the stronger the correlation.
